# ConvMut: Exploration of Viral Convergent Mutations Along Phylogenies

**DOI:** 10.3390/v18070724

**Published:** 2026-06-30

**Authors:** Tommaso Alfonsi, Anna Bernasconi, Emma Fanfoni, Cesare Ernesto Maria Gruber, Fabrizio Maggi, Daniele Focosi

**Affiliations:** 1 Department of Electronics, Information, and Bioengineering, Politecnico di Milano, 20133 Milan, Italy; tommaso.alfonsi@polimi.it (T.A.);; 2 Laboratory of Virology and Laboratories of Biosecurity, National Institute for Infectious Diseases Lazzaro Spallanzani-IRCCS, 00149 Rome, Italy; cesare.gruber@inmi.it (C.E.M.G.); fabrizio.maggi@inmi.it (F.M.); 3 Bioinformatics Research Unit in Infectious Diseases, National Institute for Infectious Diseases Lazzaro Spallanzani-IRCCS, 00149 Rome, Italy; 4 North-Western Tuscany Blood Bank, Pisa University Hospital, 56126 Pisa, Italy; daniele.focosi@gmail.com

**Keywords:** convergent evolution, convergent mutations, genomic surveillance, SARS-CoV-2, viral evolution

## Abstract

Convergent evolution in protein antigens is common across pathogens, including SARS-CoV-2; the most likely reason is the need to evade the selective pressure exerted by previous infection- or vaccine-elicited immunity. There is a pressing need for automated analysis of convergent mutations. We developed ConvMut, a tool to identify patterns of recurrent mutations in SARS-CoV-2 evolution; we exploited the granular phylogeny-based lineage hierarchy developed by PANGO, allowing us to observe *deltas*, i.e., groups of mutations that are acquired with respect to the immediately upstream tree nodes. Deltas comprise amino acid substitutions, insertions, and deletions. ConvMut can perform individual protein analysis to identify the most common single mutations acquired independently in a given subtree. Lineages are then gathered into clusters according to user-selected sets of shared mutations. An interactive graph orders the evolutionary steps of clusters, details the acquired amino acid change for each sublineage, and allows us to trace the evolutionary path until a selected lineage. ConvMut also supports frequency analysis for a given nucleotide or amino acid changes at a given residue across a selected phylogenetic subtree. ConvMut facilitates the exploration of convergent evolutionary trends in SARS-CoV-2, providing insights that could support the development of broadly effective anti-Spike monoclonal antibodies and Spike-based vaccines.

## 1. Introduction

SARS-CoV-2 (hCoV-19) is the causative agent of the COVID-19 pandemic in humans and has also been detected in multiple animal species, including several placental mammals [1]. Multiple instances of reverse zoonosis have been documented [2,3,4], leading to the establishment of animal reservoirs in which lineages that have become rare or extinct in humans may continue to circulate [5,6]. Unprecedented genome sequencing efforts throughout the world, with more than 20 million sequences that have been shared via the GISAID data science initiative [7,8,9], have shown that such massive circulation across different species creates about 29 substitutions per year, among the highest evolutionary rates observed for widely circulating human RNA viruses (e.g., measles, mumps, RSV, dengue, Zika, WNV, enterovirus D68, or influenza virus A/H3N2). From December 2019 to March 2026, more than 5600 sublineages were annotated by GISAID following PANGO designations [10], of which more than 3500 were designated after December 2021, falling under the WHO umbrella of the Omicron variant of concern (VoC) [11]. Such a granular phylogenetic tree represents a unique opportunity for studying convergent evolution. Globally, research has confirmed trends toward greater serological distance [12], translating into greater immune escape and finally into progressively higher reproductive numbers [13]. In proteomics, convergent evolution refers to the independent acquisition of genetic mutations leading to identical amino acid changes. The aim of those changes is adaptation to environmental variables, which for a pathogen implies immune evasion and survival.

Convergent evolution in SARS-CoV-2 has been reviewed in detail elsewhere [12,14] and has led, e.g., to sudden failures of most of the therapeutic anti-Spike monoclonal antibodies [15]. Its importance for immunology remains crucial [16]. To date, most insights on SARS-CoV-2 convergent evolution stem from manual work performed by volunteers on social platforms. While several automated or semi-automated systems already track recurrent or lineage-defining mutations (e.g., AutoVEM [17,18], CovMT [19], SCORPIO [20], ViruClust/VariantHunter [21,22]), no automated tool has been developed yet for online monitoring of convergent mutations. We present here ConvMut, a comprehensive software tool for the analysis of convergent evolution in SARS-CoV-2 proteins.

## 2. Methods

### 2.1. Concept Definitions

We keep note of the PANGO lineage nomenclature, which is updated regularly [10]; thanks to the hierarchical nature of PANGO nomenclature, we can completely reconstruct the implied hierarchy, focusing on the alias naming scheme that represents the full hierarchical provenance. For instance, if JN is an alias for B.1.1.529.2.86.1, then JN.1 is represented by the *unaliased* lineage B.1.1.529.2.86.1.1 in our hierarchical data structure.

For each lineage, its *constellation* is a list of all the nucleotide mutations (expressed with regard to the reference genome) that are progressively acquired by one tree node (representing either a designated or undesignated lineage) with respect to its previous node in the hierarchy. This traces a path along the tree, starting from its root to the lineage represented in that line. Different tree levels are marked with the symbol “>”; possibly, multiple substitutions are incorporated in the same level (meaning that these are acquired jointly by the observed node). As an example, the lineage B.1.617.2 can be associated with the path “C14408T > C241N > C3037T > A23403G > G29742N > T22917G > T27638C > G28881T > G210N > C21618G > G29402T > C23604G > C25469T > C22995A, C27752T > A28461G”, indicating that—starting from the root lineage designated as B—15 levels of the phylogeny are traversed. At each step on the tree, one mutation is acquired with respect to the previous node (first C14408T, then C241N, etc.). Finally, the two mutations C22995A and C27752T are acquired by the parent node of B.1.617.2 with respect to the grandparent node, whereas A28461G is acquired by B.1.617.2 with respect to its parent node.

### 2.2. Data Input Pipeline

The ConvMut application is available on GISAID EpiCoV [8], where it leverages a file (completely computed by GISAID internal pipelines) that contains lines composed of Lineage, Constellation, the earliest collection date of the lineage, and the lineage sequence count. The mutation calling procedure is 1-based and employs the EPI_ISL_402124 (WIV04) sequence as the reference genome [23]. The pipeline includes substitution, insertion, and deletion mutations. This data structure is updated daily and aligned with the full database of GISAID. Note that an open implementation is also provided on our GitHub repository [24]; here, the mutation calling procedure employs the Wuhan-Hu-1 reference genome [25].

### 2.3. Data Processing Pipeline

#### 2.3.1. Tree Reconstruction and Translation

Once the input file has been prepared, we rebuild the original parent–child and child–parent links in the phylogeny-based lineage hierarchy of SARS-CoV-2 by only including designated lineages.

We build *template genomes* for each lineage by updating the reference genome with the alternative nucleotide indicated in the mutations that are assigned to the lineage (substitutions) or shifting the sequence onward (insertions)/backward (deletions). Lineage characterizations derive from the clustering of a large number of similar sequences; therefore, ambiguous mutations are typically absent or discarded, nonetheless. Note that reversions did not produce any change here.

For each template genome, we extract the open reading frames 1a, 1b, 3a, 6, 7a, 7b, 8, 9b and the genes S, E, M, N using the common alignment tool MAFFT [26] (last available version 7.526 (April 2024) with default parametrization) and translate them into the corresponding proteins up until the first stop codon; premature stops cause shorter proteins. After alignment with the reference protein, we obtain a data structure mapping the mutated residues to the reference protein. The reference-aligned protein sequences are then useful to extract the wildtype-lineage deltas, the inter-lineage deltas, and the mutated codons.

#### 2.3.2. Inter-Lineage Deltas

For each considered lineage (*L*), we computed a *parent-delta*, i.e., the set of reference amino acids (with their position in the reference proteins) that have mutated, happening between *L* and *L*’s parent. Exceptionally, for those few lineages for which a parent was missing, we employed the first available ancestor in the dataset.

When *L* was recombinant, we computed a *delta* with respect to each of its parents. If an amino acid with its position was present in all deltas, it was inserted in the final *L*’s delta. Otherwise, we did not retain it (as we cannot rely on known breaking point coordinates for assigning a position to specific parents). We recognize that this leads to an approximation in the following cases:

1. In the observed position (pos), LP1 exhibits a1, LP2 exhibits a2 and the recombinant lineage exhibits a3. Here, we encoded this situation as a1_pos_a3.
2. In the observed position (pos), LP1 exhibits a1, LP2 exhibits a2 and the recombinant lineage exhibits a1. Even if a1 were to be considered an actual mutation (as derived from the portion of the genome inherited from LP2) this would not be recognized as a mutation, given that it is present in at least one of the parental lineages (i.e., LP1).

#### 2.3.3. WildType-Lineage Deltas

For each lineage, we computed a *wildtype-delta* by comparing the amino acid exhibited in each position of the genome with the one present in the wildtype (i.e., WIV4 [23] for GISAID implementation and Wuhan-Hu-1 [25] for the open implementation). The “delta mutations” were stored in a dictionary. We noted that, for a given lineage, its parent-delta and wildtype-delta are not necessarily the same, as the following situations may arise:

- A certain mutation is included in parent-delta but not in wildtype-delta. This corresponds to a reversion, i.e., a mutation that re-instantiates the original wildtype genome.
- A certain mutation is included in the wildtype-delta but not in the parent-delta. Here, the observed lineage has inherited a mutation from its parent.

#### 2.3.4. Quantifying Mutation Convergence Representativeness

We group lineages by the mutations that are present in their parent-deltas, thereby extracting counts of how many lineages have acquired that mutation with respect to their direct parent. These counts are used to draw a ranking among mutations; the *most convergent mutations* are those with the highest counts, as they are the most represented ones in the parent-deltas (independently of the phylogenetic tree portion where this may happen).

Note that mutations in the same position contribute equally to computed counts, e.g., considering the position *pos*, if the *L*1 lineage exhibits the mutation *a*1_*pos*_*a*2, and the *L*2 lineage the mutation *a*3_*pos*_*a*4, the occurrence count for *pos* is increased by 2.

The previously illustrated approach may introduce a possible bias as it ignores intermediate nodes that correspond to undesignated lineages. Specifically, all mutations acquired by a node in the tree that represents a non-designated lineage fall into the parent-delta of its designated offspring, thus possibly overestimating the mutation count.

### 2.4. Web Application

ConvMut is implemented as an interactive Web data application based on the open-source Python-based framework Streamlit [27], deployed as a Docker-based container [28]. It presents a landing page with documentation and three tabs targeted to *convergence analysis*, *codon exploration*, and *mutation exploration*.

The application leverages pre-computed data structures. The corresponding workflow is run daily to capture potential updates in the input files. The data structures are then pre-loaded in the application when it is started and saved as session variables so that all tabs can reuse them and to make the application more responsive.

In the following, for example purposes, we refer to ConvMut deployment on GISAID (captured in late 2025).

#### 2.4.1. Convergence Analysis

This page allows us to analyze the most frequent convergent mutations in a specific SARS-CoV-2 protein within the entire phylogenetic tree or within a portion of the phylogeny starting from a user-selected *root* lineage. The interface user experience is built as a progressive workflow where the user is asked to select values to filter the analysis (unless default values are accepted). In the following, we define each phase.

*Selection of a SARS-CoV-2 protein and root lineage*. One *protein* among all the ones in SARS-CoV-2 must be selected. Depending on this choice, only positions/mutations on the selected protein are then considered. The default protein is “Spike”.

The *root* is the starting node of the subtree considered for the downstream analysis. Note that recombinant lineages are included in the analyzed subtree when at least one of their parent lineages is included in the subtree. ConvMut provides a tabular visualization of the subtree, where each line represents a *lineage* (plus its unaliased name), *parent-delta*, and *wildtype-delta*.

Additionally, ConvMut shows a horizontal bar plot featuring the count of independent acquisitions of mutations occurring in each position of the selected protein along the selected subtree. All different mutation types occurring at a given position contribute to the global count for a given position (e.g., R346T, R346S, and S346I all contribute to the general count position 346). For example, in a subtree rooted in JN.1.11.1, featuring 733 sub-lineages (plus JN.1.11.1 itself), we find 35 lineages whose parent-delta contains a mutation occurring on 346. Reversions count as regular mutations. The bar plot can be sorted by position or number of occurrences.

*Selection of positions of interest*. The set of positions to be included in the following visualization is pre-filled by default using the positions exceeding a threshold set during the previous phase. By default, this is set to the value of the third most frequent position—should there be ties, at most 10 positions are included. Users can freely add or remove positions or change the entire set with an auto-complete component; e.g., when the analyzed protein is Spike, it is possible to restrict the analysis to specific regions, such as the receptor-binding domain (RBD: amino acid positions 319–541) or receptor-binding motif (RBM: amino acid positions 438–506).

We term *SP* the set of positions selected by the user; we compute and show (1) a series of pie plots representing the variability of different amino acid residues in each position in *SP* and (2) a table including only the lineages within the selected subtree that include at least a position within *SP*. The table features the (aliased) lineage name, the unaliased lineage name, the cluster it belongs to, the mutations acquired by lineages in the cluster, and a string explaining how the cluster composition was computed.

For a meaningful, well-organized visualization of the map, we *cluster* lineages. Specifically—given a lineage *L*, its parent-delta, and its wildtype-delta—*L*’s corresponding cluster is computed by taking only the positions in *SP* that are included in either *L*’s parent-delta or *L*’s wildtype-delta (or both), according to Equation (1):

*cluster* = (*parent_delta* ∪ *wildtype*_*delta*) ∩ *SP* (1)

For instance, when *SP* = {346, 456}, *L*1’s parent-delta = {F456L, S680I}, and *L*1’s wildtype-delta = {S31-, R346S, F456L}, *L*1’s cluster is characterized by {346, 456}, then labelled c_346_456. Instead, with *L*2’s parent-delta = {F456L}, and *L*2’s wildtype-delta = {Q480T}, *L*2’s cluster would be characterized by {456}, then labelled simply c_456.

Note that lineages grouped in the same *cluster* do not necessarily have the same mutations with respect to the user-selected root; instead, they must exhibit the same convergent mutations (even because of a reversion process).

*Convergent mutations map*. ConvMut finally plots a map to represent the complete convergent mutation landscape of the selected protein in the selected subtree, including only mutations corresponding to the positions chosen as described above. Nodes in the map are then a specific set of PANGO-designated lineages, termed *LV* (determined as those whose parent-delta sets contain at least one mutation included in the positions of *SP*), and edges represent a parent–child (or ancestor–child) relationship between two lineages, labeled with the mutation(s) contained in the *parent-delta* of the child node. Note that the graph is developed and visualized from left to right, by representing the root and older lineages in the left-most portion of the page and the leaves (most recent lineages) in the right-most portion.

We note that two nodes that are connected by an edge in the graph are not necessarily parent/child, as—given a child—it could happen that its direct parent is not contained in *LV* (and, in turn, its grandparent, etc.). Then, edges are drawn between a node and its closest PANGO-designated ancestor included in *LV*.

We also note that some clusters may enclose nodes whose incoming edges have different labels (in the alternative amino acid residue or even in the position); this is consistent with the definition of a cluster, which gathers lineages that have the same mutations in their parent-delta or wildtype-delta, making the stage at which a mutation was acquired irrelevant.

#### 2.4.2. Codon Exploration

This page allows us to explore mutations that occur in a specific codon of the selected protein. Starting from the amino acid residue, we provide information on the determinant codon. Users input a SARS-CoV-2 protein, to be understood as a root of a phylogeny-based structure (in the PANGO lineage hierarchy) from which the analysis is produced, and a *position of interest* (among pre-filtered options only including those that are present in at least one *delta* in the selected subtree). Users can choose to visualize results based on codons or amino acid residues.

#### 2.4.3. Mutation Exploration

This page allows users to investigate a given position on a selected protein while also considering its phylogenetic background. Consistently with the previous tab, we require a protein, root lineage, and position *P* to be inspected.

We identify with *LM* all the lineages in the considered subtree that include a mutation in the *P* position in their parent-delta. The user can visualize a tree-like representation of the initial lineages’ subtree, where we prune all branches that do not present any lineage in *LM*; we also collapse tree levels not containing any lineage (or parent of a lineage) in *LM*. Mutation labels are colored to make differences in amino acids more apparent.

## 3. Results

**Figure 1:**
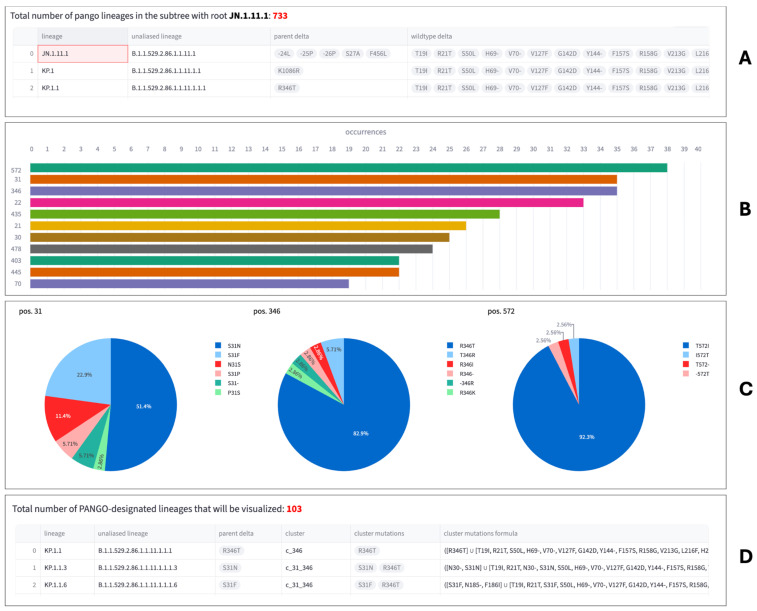
Convergence analysis: intermediate result visualization. (**A**) A table with all the lineages that derive from the chosen root (JN.1.11.1, in the example); (**B**) a bar plot that, for each position on the selected protein, counts how many lineages independently acquired a mutation in that position, sorted by count; (**C**) pie plots that capture the variability of different amino acid residues in the three selected positions (i.e., those above the chosen threshold, 35, according to the bar plot in panel (**B**); (**D**) a table detailing a lineage’s cluster name and its mutations for each of the lineages to be plotted in the graph.

In this section, we report sample results for each of the ConvMut main functions. Figure 1 shows intermediate results for the *convergence analysis* when the following inputs are selected: protein = S (Spike), root = JN.1.11.1, threshold = 35 (to include positions of interest), and selected positions (from more represented to less represented) = 346, 31, 572. Panel A illustrates a table with lineages (normal and unaliased) described by their parent-delta and wildtype-delta; Panel B shows a bar plot whose *y*-axis represents positions along the chosen protein and *x*-axis represents the count of lineages acquiring a mutation in that position; Panel C shows pie plots for each of the positions extracted above the set threshold (along the bar plot shown in B), showing how mutations are distributed by alternative amino acids; Panel D presents a table of lineages to be visualized in the graph, detailing their clusters, the clusters’ specific mutations and how these were computed (see Equation (1) in *Methods*).

**Figure 2:**
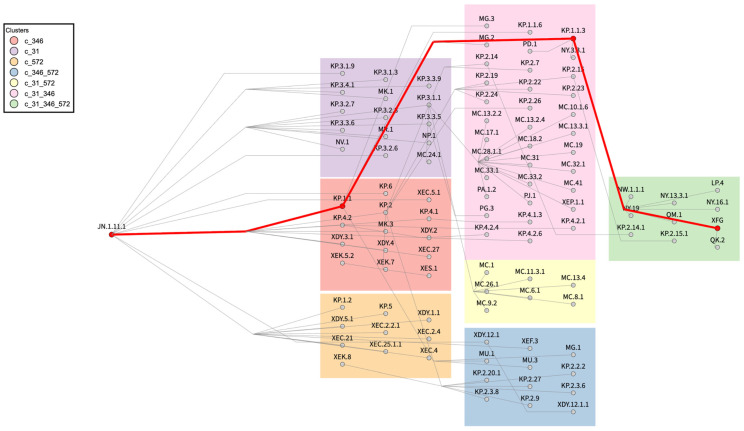
ConvMut convergence analysis tree. This example captures the case considering in the input Spike mutations 346, 31, 572 in the JN.1.11.1 subtree. The path leading from the root JN.1.11.1 to the recent recombinant lineage XFG is highlighted in red.

As a result of the previous parameters’ selection, a graph is rendered as shown in Figure 2. By selecting a specific lineage in the dropdown menu above the graph, we can highlight the specific relevant path of converging events on the subtree. For example, in Figure 2, we show the path of XFG, a recombinant lineage that is of particular interest at the time of writing [29], obtained by exploiting the features of the implemented interactive visualization. Specifically, the chain to be followed from the root (left) to the selected lineage (right) can be described as follows: JN.1.11.1 acquired R346T in KP.1.1, which acquired S31N in KP.1.1.3. This, in turn, acquired T572I in NY.19, finally possibly corresponding to the triplet < P31S, T346R, I572T> in XFG.

Note that XFG derives from LP.8.1.2 (acceptor) and LF.7 (donor), with a breakpoint around nt 1507 of the Spike protein [30]; according to the limitations explained in Section 2.3.2, in ConvMut we cannot rely on automatically computing breakpoint coordinates to assign a position to specific parents. As a result, in this map, we do not definitely determine whether the mutations in positions 21, 346 and 572 come from the LP.8.1.2- or LF.7-derived chains.

**Figure 3:**
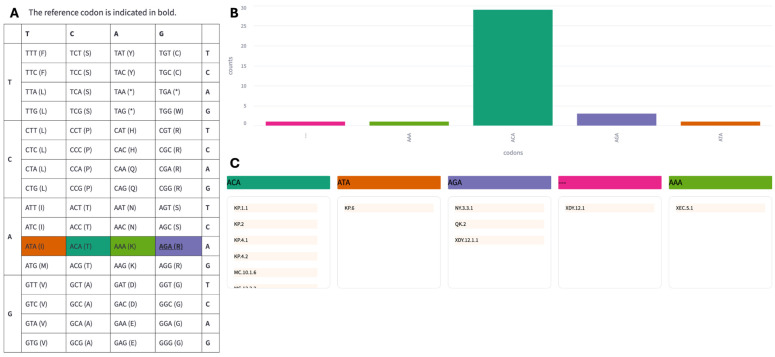
ConvMut codon exploration view. This example corresponds to the input selection JN.1.11.1 (root) and 346 (position): (**A**) shows the genetic code table; (**B**) overviews a bar plot of counts of mutations, and (**C**) lists lineages corresponding to each codon.

Then, Figure 3 provides an overview of the possible output of the *codon exploration* analysis when input root = JN.1.11.1 and pos = 346 are selected. Panel A shows the genetic code (correspondences between nucleotide triplets and the corresponding amino acid residues) where we underline the codon exhibited in the wildtype genome in the position of interest *p* and color-highlight the codon present in that position in those lineages that feature an amino acid change in *p* and their parent-delta. Panel B shows the counts of mutations per type of codon, i.e., the number of lineages exhibiting that codon in their parent-delta, in the selected position. Panel C details the lineages contributing to those counts.

**Figure 4:**
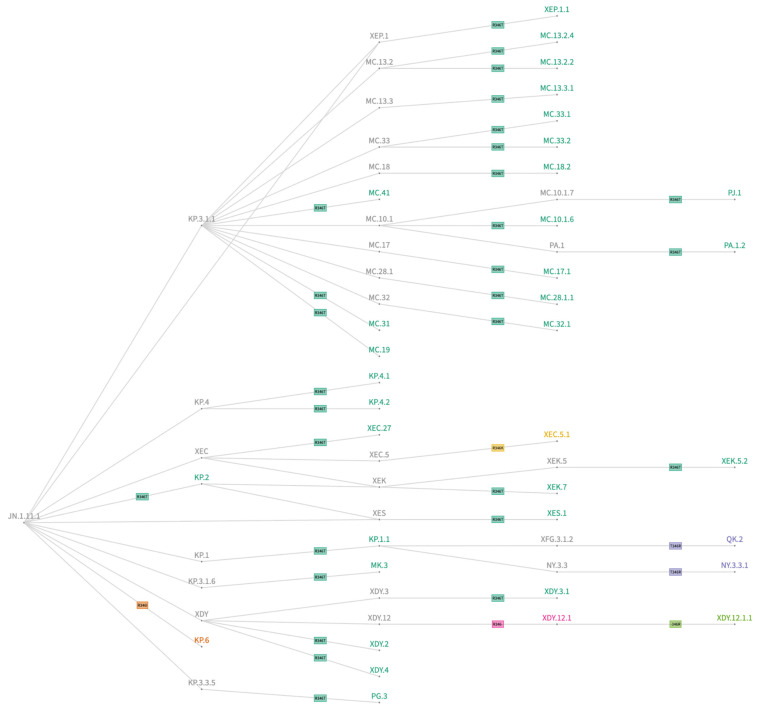
ConvMut mutation exploration tree. This example corresponds to the input selection with root JN.1.11.1 and position 346. Labels on edges indicate the different mutations that occurred at each position and were acquired by the node on the right of the edge. Note that we use different colors to mark different exhibited amino acid residues.

Finally, Figure 4 provides the visualization built within the *mutation exploration* tab for root = JN.1.11.1 and Spike protein position 346.

### A Posteriori Validation

A group of authors of this manuscript previously manually generated convergence maps which were published in two reviews in early 2023 [31] and mid 2024 [14] respectively.

Specifically, these maps focused on SARS-CoV-2 Omicron sublineages (e.g., BA.2 and BA.4/5, with focuses on CZ.1, CH.1.1.1, and other BA.2.75.* descendants) [31], XBB.* sublineages, JN.1* sublineages [14], and FLip’s mutations (documented in [32]).

To validate ConvMut, we conducted a manual confirmation of all these maps by carefully comparing the output of the automatic implementation of ConvMut with one of the maps. To do so, we appropriately ran the system “a posteriori” on snapshots reflecting the status of the PANGO-phylogeny-based lineage hierarchy at the time when the manual maps were drafted. Slight adjustments were necessary to consider the contingency of newly proposed lineage assignments and rapid modifications of the nomenclature. Overall, we were able to reconstruct and confirm a large part of the manual observations, confirming a considerable gain in automatizing the initially cumbersome and error-prone process of map generation.

## 4. Discussion

The analysis of convergent evolution in a pathogen is of paramount importance to predicting the changing landscape where antiviral drugs have to act. Clearly, convergent evolution is more evident for protein antigens that are targeted by the selective pressure of the immune response, either infection- or vaccine-elicited. Throughout the six years of the COVID-19 pandemic, the clearest example of convergent evolution has been within the Spike RBD: it has happened in waves and has led to dramatic failures of recently authorized therapeutic anti-Spike monoclonal antibodies [15]. With the support of ConvMut we have also been able to seamlessly identify N-terminal domain evolution [33]. While most of the effort has historically been on studying divergence in order to provide granular phylogenetic trees, the myriad of sublineages identified for SARS-CoV-2 have posed challenges for the classification of variants. Scientists in the community have volunteered to keep track of shared sets of Spike mutations regardless of their origin and to represent charts that could be easily interpreted by both the general public and vaccine/antibody manufacturers.

Our study has several limitations. First, ConvMut is dependent on the PANGO nomenclature [10], whose designation criteria are somewhat arbitrary; although it is the most widely adopted, currently, this nomenclature does not necessarily correspond to robust phylogenetic clades. While ConvMut is agnostic to supporting different input phylogenies, currently, the only complete and publicly accessible, up-to-date data source is the one embedded and fed through GISAID pipelines. Second, for recombinant lineages, ConvMut is not able to discriminate the parental source of a given convergent mutation, but—in many instances—the exact breakpoint has not been discriminated against. Third, when estimating the commonest convergent mutations, ignoring the intermediate PANGO-undesignated nodes could lead to an overestimate of mutation counts; the methodology relies completely on the quality of sequencing data, phylogenetic reconstruction, sampling or mutation rates, which are outside of our control.

The strengths of ConvMut are: (1) the uniqueness of each of its tools among the plethora of SARS-CoV-2 bioinformatics tools generated so far; (2) the automated updates to GISAID, which is currently the most timely data source for SARS-CoV-2 genomes, lineage assignment and counts; (3) its availability as a user-friendly graphical interface; and (4) the possibility to export the generated charts.

ConvMut could prove useful at forecasting trends in SARS-CoV-2 evolution, not just benefiting basic virological research but also informing drug and vaccine development. By highlighting recurrent mutational trends, it may help prioritize candidate targets and support decision making during early-stage development. This is increasingly important in light of the recent developments in immunobridging [34], which can shorten the time from drug design to market authorization. The architecture of ConvMut is amenable to application to viral pathogens other than SARS-CoV-2 whenever granular phylogenies are available.

## 5. Conclusions

As SARS-CoV-2 continues to evolve, the same mutations often appear independently in different viral lineages, helping the virus evade immunity and undermining vaccines and antibody therapies. While tracking these repeated changes across millions of viral genomes has thus far largely relied on manual, fragmented efforts, ConvMut stands as an automated interactive platform for identifying and visualizing recurrent mutations across the viral family tree, enabling researchers to pinpoint the most common evolutionary hotspots.

Most importantly, by making convergent evolution easier to detect and interpret, this approach may inform the design of vaccines and antibody treatments by identifying recurrent mutational trends relevant to viral evolution. Embedding ConvMut within the GISAID framework provides a completely up-to-date instance at the service of immunologists and practitioners around the world.

From a more general perspective, ConvMut is an additional tile in our long-running contribution to SARS-CoV-2 data-driven analysis and global surveillance in the context of a complex data ecosystem, recently focusing on database-wide analysis of mutation fixation or cyclical reversion across time [35], the detection of recombination patterns [36,37], and the identification of fast-emerging variants [21,22]. Altogether, this tool ecosystem supports more informed health responses worldwide, as a follow-up to the “SENSIBLE” project vision [38]. Specifically, ConvMut’s model could be readapted to different emerging human pathogens for which granular phylogenies are available, contributing to designing strategies to combat diseases different from COVID-19.

## Data Availability

The dataset analyzed during the current study corresponds to the full SARS-CoV-2 database of GISAID and is available on the www.gisaid.org platform, EpiCoV repository, corresponding to 17,632,937 sequences as of 28 May 2026 (time of submission of this manuscript); these can be downloaded by registered users through any institutional account. The ConvMut software is usable on GISAID for all users with platform registration. An open implementation of the software is available at https://github.com/DEIB-GECO/open-ConvMut [24] (accessed on 28 May 2026).

## Abbreviations

The following abbreviations are used in this manuscript:

RSV	Respiratory Syncytial Virus
WNV	West Nile Virus
GISAID	Global Initiative on Sharing All Influenza Data
VoC	Variant of concern
SARS-CoV-2	Severe Acute Respiratory Syndrome Coronavirus 2
MAFFT	Multiple Alignment using Fast Fourier Transform
RBD	Receptor-binding domain
RBM	Receptor-binding motif
